# Trombose de Aorta e Artéria Renal como Manifestação Clínica Inicial da COVID-19 em um Receptor de Transplante Cardíaco

**DOI:** 10.36660/abc.20201210

**Published:** 2021-11-01

**Authors:** Deborah de Sá Pereira Belfort, Fabiana G. Marcondes-Braga, Sandrigo Mangini, Caio Rebouças Fonseca Cafezeiro, Diógenes Amauri Gonçalves Furlan, Fernando Bacal

**Affiliations:** 1 Universidade de São Paulo Faculdade de Medicina Hospital das Clínicas São Paulo SP Brasil Instituto do Coração (InCor), Hospital das Clínicas da Faculdade de Medicina da Universidade de São Paulo, São Paulo, SP - Brasil

**Keywords:** COVID-19, Tromboembolia, Transplante de Coração

## Introdução

A nova infecção pelo coronavírus surgiu em Wuhan, na China, no final do ano de 2019, e é hoje uma pandemia.^1^ A relação da COVID-19 com eventos trombóticos já está bem estabelecida, mesmo em pacientes em anticoagulação profilática. Embora eventos tromboembólicos arteriais e venosos tenham sido descritos, principalmente acidente vascular cerebral e infarto agudo do miocárdio (IAM),^2,3^ existem poucos relatos de trombose arterial em locais incomuns.^4^ Quase todos os relatos são de eventos trombóticos ocorridos em pacientes internados em unidade de terapia intensiva (UTI), e a incidência de tromboembolismo em casos moderados de COVID-19 ainda não está clara.

Nós apresentamos um caso de um receptor de transplante cardíaco, do sexo masculino, admitido no departamento de emergência apresentando trombose da artéria renal direita e da aorta torácica descendente associada com COVID-19.

### Apresentação do caso

Paciente de 28 anos de idade, do sexo masculino, receptor de transplante cardíaco desde 2018, com história de cardiomiopatia dilatada familiar, foi admitido no departamento de emergência com dor aguda no flanco direito por três dias, associada a febre, calafrios, náusea e vômitos. O paciente negou sintomas respiratórios, mialgia, cefaleia, ou outros sintomas que poderiam sugerir infecção viral. Além de diabetes mellitus e dislipidemia, o paciente não apresentava nenhuma outra comorbidade. O paciente estava em uso regular de tacrolimo, micofenolato e prednisona.

O exame físico revelou pressão arterial de 150/100 mmHg, frequência cardíaca de 100 bpm, taxa respiratória de 20 ciclos por minuto, e saturação de oxigênio sanguíneo de 96% em ar ambiente. Não foram detectados ruídos respiratórios na avaliação pulmonar, e o exame abdominal revelou sensibilidade no ângulo costovertebral. Os exames de sangue mostraram níveis de proteína C reativa de 317mg/dL, lactato desidrogenase de 1827U/L, D-dímero de 4126ng/mL, ferritina de 651ng/mL, leucócitos de 16100/mm^3^ e nenhuma outra alteração.

**Figura 1:**
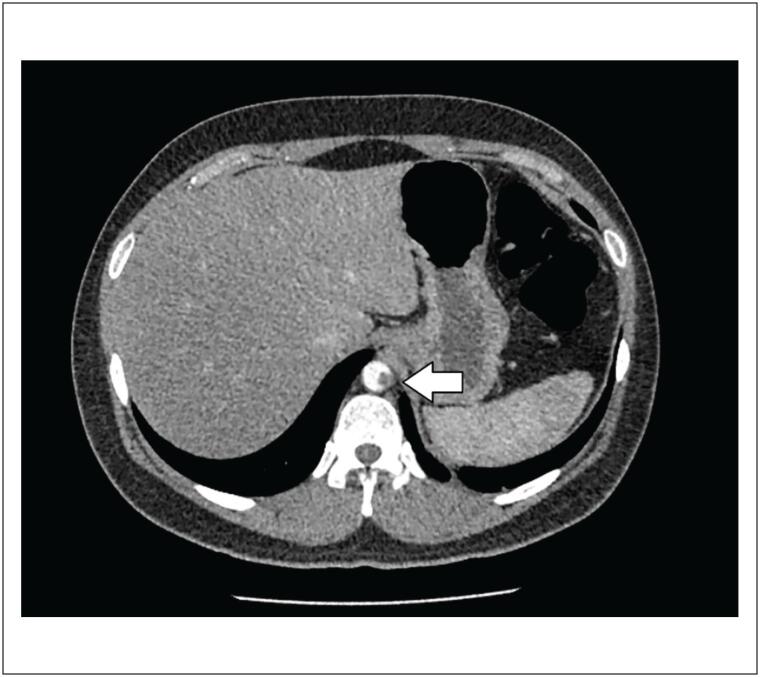
Tomografia computorizada abdominal revelando trombose luminal periférica esparsa na aorta torácica descendente (seta).

**Figura 2:**
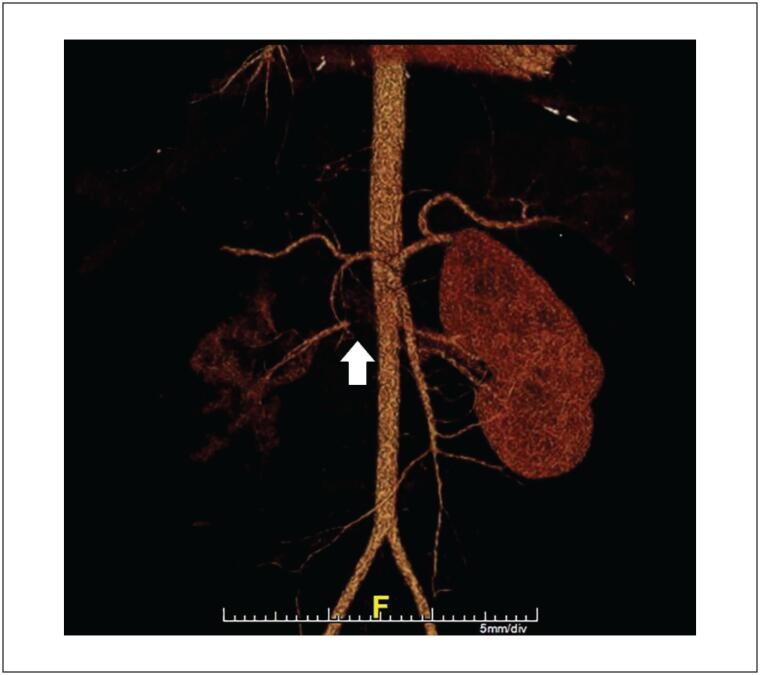
Reconstrução tridimensional da aorta abdominal mostrando suboclusão do segmento proximal da artéria renal direita.

**Figura 3:**
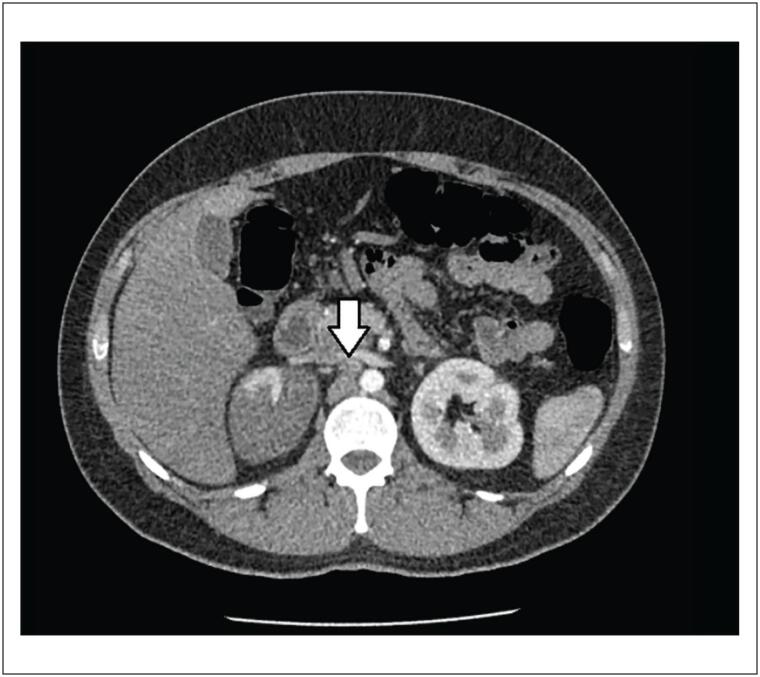
Tomografia computorizada abdominal mostrando um dos trombos se estendendo até o óstio da artéria renal direita, causando oclusão parcial do segmento proximal da artéria (seta). Rim direito com áreas hipodensas compatíveis com infarto renal.

**Figura 4:**
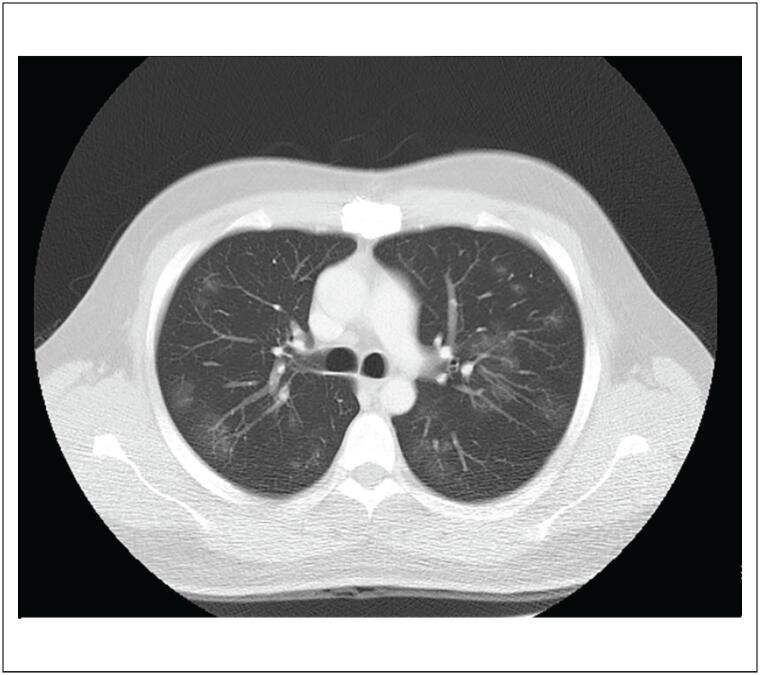
Tomografia computadorizada de tórax apresentando opacidade em vidro fosco em 25% do parênquima pulmonar.

Tomografia computorizada (TC) abdominal e de tórax revelou trombose luminal periférica esparsa na aorta torácica descendente (Figura 1). Um dos trombos se estendeu até o óstio da artéria renal direita, causando oclusão parcial do segmento proximal da artéria (Figuras 2 e 3). O rim direito apresentou áreas hipodensas compatíveis com infarto renal (Figura 3). Nenhuma outra artéria foi afetada. Além desses achados, opacidade em vidro fosco foi detectada em 25% do parênquima pulmonar (Figura 4) e, por essa razão, suspeitou-se de COVID-19. O método de reação em cadeia da polimerase fluorescente em tempo real (teste nasofaríngeo) revelou resultado positivo para SARS-Cov-2. Testes de coagulopatia foram realizados antes de se iniciar a anticoagulação. Níveis de proteína C, proteína S, a antitrombina III foram normais, o teste de mutação da protrombina foi negativo, os testes de detecção de anticorpos anticardiolipina (aCL) (IgG e IgM) foram negativos, e o teste de detecção de anticoagulante lúpico foi positivo.

Foram prescritos hidratação, antibióticos (ceftriaxona e azitromicina), e anticoagulação com enoxaparina. Tacrolimo e micofenolato foram interrompidos, e a prednisona substituída por hidrocortisona 150 mg/dia na admissão. O paciente apresentou melhora e ficou assintomático. Marcadores inflamatórios diminuíram nos dias seguintes. A imunossupressão foi reiniciada após cinco dias de admissão, e prescrita varfarina. O paciente recebeu alta no 15º de internação após ajuste da dose de varfarina.

## Discussão

Desde o surto da COVID-19, uma variedade de apresentação clínica tem sido descrita. A maioria dos pacientes apresentam sintomas leves, mas até 14% dos pacientes infectados desenvolvem pneumonia intersticial, e 5% necessitam de ventilação mecânica.^1^ Eventos tromboembólicos em pacientes críticos foram associados com COVID-19 em vários estudos.^2-4^

Os mecanismos de distúrbios trombóticos e das coagulopatias não foram totalmente esclarecidos. A COVID-19 está associada a um estado pró-inflamatório, e a tempestade de citocinas descrita na COVID-19 contribui para trombose por meio da ativação de monócitos, neutrófilos e endotélio.^4^ Essas células ativam plaquetas e aumentam os níveis de fator de von Willebrand e fator VIII, que contribuem para a geração de trombina e formação de coágulo de fibrina. A trombina, por outro lado, amplifica as vias pró-inflamatórias.^5^ O vírus pode ainda causar endotelite pelo receptor de enzima conversora da angiotensina 2, levando a microangiopatia trombótica.^6^

Apesar de a doença grave provocar um estado de hipercoagulabilidade, eventos tromboembólicos podem ocorrer em ambientes ambulatoriais, o que reforça que a doença crítica não é o único fator envolvido. Overstad et al. relataram tromboembolismo venoso (TEV) em quatro pacientes em isolamento domiciliar,^7^ e um estudo na Itália mostrou que 50% dos eventos tromboembólicos foram diagnosticados nas primeiras 24 horas de internação.^8^

Eventos tromboembólicos arteriais, apesar de menos comuns que TEV, ocorrem em até 10,5% dos pacientes hospitalizados.^2^ Acidente vascular cerebral foi descrito em 1,6% a 3,8% dos pacientes com COVID-19,^2,4^ enquanto a incidência de infarto agudo do miocárdio variar de 1,1%4 na Itália a 8,9% em diferentes centros de Nova Iorque.^2^ Locais incomuns de ocorrência de trombose também são descritos. Acroisquemia e isquemia nos membros foram descritos em um caso associado com múltiplos infartos cerebrais.^9^ Isquemia intestinal foi detectada em uma mulher com insuficiência respiratória aguda trombose da veia porta direita e trombose da veia mesentérica superior na admissão.^10^ Dois casos de infarto renal foram relatados por Post et al.,^11^ um deles em um receptor de transplante renal, e ambos internados na UTI.

Há poucos relados de casos de COVID-19 envolvendo receptores de transplante cardíaco. Uma série de casos em Nova Iorque relatou mortalidade de 25%, mas nenhum caso de evento tromboembólico foi descrito.^8^ Nós descrevemos aqui o primeiro caso de trombose arterial em um paciente submetido a transplante cardíaco.

Devido à apresentação atípica, buscamos por uma trombofilia subjacente, e encontramos resultado positivo no teste de detecção de anticoagulante lúpico. Foi relatada associação da COVID-19 com resultado positivo para anticorpos antifosfolipídios (AA). Zhang et al.^9^ descreveram três casos de trombose associada com AA, representada por aCL e anti-β2-glicoproteína I (aβ2GPI), mas não foi detectado anticoagulante lúpico em nenhum dos pacientes.^9^ Por outro lado, Harzallah et al.^12^ relataram positividade para o anticoagulante lúpico em 45% dos 56 pacientes, e teste positivo para aCL ou aβ2GP somente em 10% dos pacientes, a maioria associado com aCL.^12^ Contudo, sabe-se que infecções agudas são sabidamente associadas com AA positivos.^13^ Por isso, a importância da positividade para AA na COVID-19 precisa ainda ser determinada.

## Conclusão

Este relato de caso ilustra a heterogeneidade da apresentação clínica da COVID-19, e reforça a existência de um estado pró-trombótico, mesmo em ambiente ambulatorial. Ainda, este estudo contribui com informações sobre a presença de AA na COVID-19, apesar que sua importância na fisiopatologia dos eventos tromboembólicos ainda não ter sido definida nesse cenário. A implicação desses achados nos pacientes transplantados é ainda menos clara, e este relato de caso destaca a necessidade de mais pesquisas.
