# Supplementary material for: Trombose de Aorta e Artéria Renal como Manifestação Clínica Inicial da COVID-19 em um Receptor de Transplante Cardíaco
Source: Arq Bras Cardiol. 2021 Nov 1;117(5):1045–7. [Article in Portuguese] doi: 10.36660/abc.20201210 (PMC8682100; doi:10.36660/abc.20201210)
Supplement: Translation

# Translation: Aortic and Renal Artery Thrombosis as the First Clinical Manifestation of COVID-19 in a Heart Transplant Recipient

**Authors:** Deborah de Sá Pereira Belfort, Fabiana G. Marcondes-Braga, Sandrigo Mangini, Caio Rebouças Fonseca Cafezeiro, Diógenes Amauri Gonçalves Furlan, Fernando Bacal

**Affiliations:** 1 São Paulo SP Brazil Instituto do Coração (InCor), Hospital das Clínicas da Faculdade de Medicina da Universidade de São Paulo, São Paulo, SP - Brazil

**Keywords:** COVID-19, Thromboembolism, Heart Transplantation

## Introduction

The novel coronavirus infection emerged in Wuhan, China, in the end of 2019 and is now a pandemic.^1^ The relation between COVID-19 and thrombotic events is well established, even for patients under prophylactic anticoagulation. Although venous and arterial thromboembolic events have been described, usually stroke and acute myocardial infarction (AMI),^2,3^ there are few reports of arterial thrombosis in unusual sites.^4^ Almost all reports of thrombotic events are of intensive care unit (ICU) patients, and the incidence of thromboembolism in mild cases of COVID-19 is still not clear.

We report a case of a male heart transplant recipient admitted to emergency department with thrombosis of right renal artery and thoracic descending aorta associated with COVID-19.

### Case presentation

A 28-year-old male heart transplant recipient since 2018, with previous familial dilated cardiomyopathy was admitted to the emergency department with acute right flank pain for three days associated with fever, chills, nausea, and vomiting. He denied respiratory symptoms, myalgia, headache or other symptoms which could suggest viral infection. Apart from diabetes mellitus and dyslipidemia, he had had no other comorbidity. The patient was on regular use of tacrolimus, mycophenolate and prednisone.

Physical exam revealed blood pressure of 150/100 mmHg, heart rate of 100 beats per minute, respiratory rate of 20 cycles per minute and blood oxygen saturation of 96% in ambient air. No rales were detected in lung evaluation and abdominal exam showed right costovertebral angle tenderness. Blood tests showed C-reactive protein of 317mg/dL, lactate dehydrogenase of 1,827U/L, D-dimer of 4,126ng/mL, ferritin of 651ng/mL, leukocytosis of 16,100/mm^3^ and no other alterations.

An abdominal and thoracic computed tomography scan (CT scan) was performed and, surprisingly, revealed sparse luminal peripheral thrombi in the descending thoracic aorta (Figure 1). One of the thrombi extended to the right renal artery ostium and caused partial occlusion of its proximal segment (Figures 2 and 3). Right kidney presented multiple hypodense areas compatible with renal infarcts (Figure 3). No other artery was affected. Besides those findings, ground-glass opacities were found in 25% of pulmonary parenchyma (Figure 4), and for this reason COVID-19 was suspected. Nasopharynx real-time fluorescence polymerase chain reaction result for SARS-CoV-2 was positive. Coagulopathy tests were performed before starting anticoagulation. Protein C, protein S, antithrombin III levels were normal, prothrombin mutation test was negative, anticardiolipin (acL) antibody (IgG and IgM) tests were negative, but lupus anticoagulant (LAC) test was positive.

**Figure 1:** Abdominal computed tomography scan revealing sparse luminal peripheral thrombi in the descending thoracic aorta (arrow).

**Figure 2:** Tridimensional reconstruction of abdominal aorta showing partial occlusion of the proximal segment of right renal artery.

**Figure 3:** Abdominal computed tomography scan showing one of the thrombi extending to right renal artery ostium and causing partial occlusion of the proximal segment of this artery (arrow). Right kidney presents hypodense areas compatible with renal infarcts.

**Figure 4:** Thoracic computed tomography scan revealing ground-glass opacities in 25% of pulmonary parenchyma.

Hydration, antibiotics (ceftriaxone and azithromycin) and anticoagulation with enoxaparin were prescribed. Tacrolimus and mycophenolate were discontinued and prednisone was switched to hydrocortisone 150mg/day at admission. The patient improved and became asymptomatic. Inflammatory markers went down the following days. Immunosuppression was restarted after five days of admission and warfarin was prescribed. The patient was discharged on the 15^th^ hospitalization day after adjustment of warfarin dose.

## Discussion

Since the outbreak of COVID-19, a wide range of clinical presentations have been described. Most patients have mild symptoms, but up to 14% of infected patients develop interstitial pneumonia and 5% require mechanical ventilation.^1^ Thromboembolic events in critically ill patients have been associated with COVID-19 in several studies.^2-4^

The mechanisms of the prothrombotic state and coagulopathy are not totally clear. COVID-19 is associated with a marked proinflammatory state, and the cytokine storm described in COVID-19 contributes to thrombosis by activating monocytes, neutrophils, and endothelium.^4^ These cells activate platelets and increase levels of von Willebrand factor and factor VIII, all contributing to thrombin generation and fibrin clot formation. Thrombin, on the other hand, amplifies pro-inflammatory pathways.^5^ The virus may also cause endotheliitis through the angiotensin-converting enzyme 2 receptor, leading to thrombotic microangiopathy.^6^

Although severe illness itself is known to provoke a hypercoagulable state, thromboembolic events may happen in outpatient settings, highlighting that critical illness is not the only factor involved. Overstad et al. reported venous thromboembolism (VTE) in four patients in isolation at home,^7^ and a study in Italy showed that 50% of the reported thromboembolic events were diagnosed within 24h of hospital admission.^8^

Arterial thromboembolic events, although less common than VTE, occur in up to 10.5% of hospitalized patients.^2^ Stroke was described in 1.6% and 3.8% of patients with COVID-19,^2,4^ while the incidence of acute myocardial infarction (AMI) varies from 1.1%^4^ in Italy to 8.9% in different centers of New York City.^2^ Unusual sites of arterial thrombosis are also reported. Limb or acral ischemia was described in a case associated with multiple brain infarcts.^9^ Bowel ischemia was detected in a woman presenting acute respiratory failure and right portal vein and upper mesenteric thrombosis on admission.^10^ Two cases of renal infarction were reported by Post et al.,^11^ one of them in a kidney transplant recipient, and both patients were in ICU.

There are few reports of COVID-19 cases among heart transplant recipients. A series of case in New York reported mortality of 25%, but no cases of arterial thromboembolic events have been described.^8^ We report here, the first case of arterial thrombosis in a heart transplant patient.

Because of the atypical presentation, we searched for an underlying thrombophilia, and we found a positive LAC. It has been reported association of COVID-19 with positivity of antiphospholipid antibodies (AA). Zhang et al.^9^ described three cases of thrombosis associated with AA represented by aCL and anti-β2-glycoprotein I (aβ2GPI), but no LAC was detected in any of the patients.^9^ On the other hand, Harzallah et al.^12^ reported positivity of LAC in 45% of 56 patients, and only 10% positive for aCL or aβ2GPI, most of them associated with LAC.^12^ However, acute infections are known to be sometimes associated with transient AA.^13^ For those reasons, the relevance of AA positivity in COVID-19 is yet to be determined.

## Conclusions

This case report illustrates the heterogeneity of clinical presentation of COVID-19 and reinforces the existence of a prothrombotic state, even in the outpatient setting. Moreover, it adds information to the recent reports regarding the presence of AA in COVID-19, although their importance in the pathophysiology of thromboembolic events in this setting is still not defined. The implication of these findings in transplant patients is even less clear, and this case report highlights the need for further research.
